# SEIR epidemic model for COVID-19 transmission by Caputo derivative of fractional order

**DOI:** 10.1186/s13662-020-02952-y

**Published:** 2020-09-14

**Authors:** Shahram Rezapour, Hakimeh Mohammadi, Mohammad Esmael Samei

**Affiliations:** 1 Institute of Research and Development, Duy Tan University, Da Nang, 550000 Vietnam; 2 Faculty of Natural Sciences, Duy Tan University, Da Nang, 550000 Vietnam; 3 Department of Medical Research, China Medical University Hospital, China Medical University, Taichung, Taiwan; 4 Department of Mathematics, Miandoab Branch, Islamic Azad University, Miandoab, Iran; 5 Department of Mathematics, Bu-Ali Sina University, 65178 Hamedan, Iran

**Keywords:** 34A08, 65P99, 49J15, COVID-19, Equilibrium point, Numerical simulation, SEIR model

## Abstract

We provide a SEIR epidemic model for the spread of COVID-19 using the Caputo fractional derivative. The feasibility region of the system and equilibrium points are calculated and the stability of the equilibrium points is investigated. We prove the existence of a unique solution for the model by using fixed point theory. Using the fractional Euler method, we get an approximate solution to the model. To predict the transmission of COVID-19 in Iran and in the world, we provide a numerical simulation based on real data.

## Introduction

Coronaviruses are crown viruses that can cause disease in humans and animals. In humans, several coronaviruses are known to cause respiratory illnesses such as common cold and more severe illnesses such as acute Middle East respiratory syndrome (SARS), severe acute respiratory syndrome (SARS), and a recently discovered disease COVID-19. A coronavirus (COVID-19) that was first identified in the Chinese city of Wuhan in 2019 is a new strain that has not been previously identified in humans. Snakes or bats have been suspected as a potential source for the outbreak, though other experts currently consider this to be unlikely. Fever, cough, shortness of breath, and breathing difficulties are initial symptoms of this infection. In the next steps, the infection can cause pneumonia, severe acute respiratory syndrome, kidney failure, and even death.

The study of disease dynamics is a dominating theme for many biologists and mathematicians (see, for example, [1–12]). In recent years, many physical and biological problems have been modeled by fractional-order derivatives. The main reasons for using a fractional-order system (FDE) is related to systems with memory, history, or nonlocal effects which exist in many biological systems that show the realistic biphasic decline behavior of infection or diseases but at a slower rate. It has been studied by many researchers that fractional extensions of mathematical models of integer order represent the natural fact in a very systematic way such as in the approach of Etemad et al. [13–15], Hedayati et al. [13, 16–19], Baleanu et al. [11, 18, 20, 21], and Mahdy et al. [22, 23]. In recent years, many papers have been published on the subject of Caputo–Fabrizio fractional derivative (see, for example, [24–30]). Mathematical models are used to simulate the transmission of coronavirus (see, for example, [31–37]).

In this paper, we intend to investigate the spread of COVID-19 disease using the SEIR mathematical model with the Caputo fractional-order derivative. First, we analyze the model mathematically and then, in the numerical section, we present simulations for the release of COVID-19 in Iran and around the world. Also, to evaluate the advantage of using the fraction derivative, we make a comparison between the results of the model with the fractional- and integer-order derivative with the real data to determine which one provides a better approximation in this model. By calculating the model results for different orders of fractional derivative, we investigate the effect of derivative order on the behavior of the resulting functions and equilibrium points and resulting numerical values.

The structure of the paper is as follows. In Sect. 2 some basic definitions and concepts of fractional calculus are recalled. The SEIR model of fractional order for COVID-19 transmission is presented in Sect. 3, and the equilibrium points and the reproduction number are calculated. The stability of the equilibrium points is analyzed in Sect. 4. The existence and uniqueness of solution for the system is proved in Sect. 5. In Sect. 6, a numerical method for solving the model is described and a numerical simulation is presented.

## Preliminary results and definitions

In this section, we recall some of the fundamental concepts of fractional differential calculus, which are found in many books and papers.

### Definition 1

([38])

For an integrable function *g*, the Caputo derivative of fractional order $\nu \in (0,1)$ is given by

$$ {}^{C}D^{\nu }g(t)=\frac{1}{\Gamma (m-\nu )} \int _{0}^{t} \frac{g^{(m)}(\upsilon )}{(t-\upsilon )^{\nu -m+1}} \,d\upsilon , \quad m=[\nu ]+1. $$

Also, the corresponding fractional integral of order *ν* with $Re (\nu ) > 0$ is given by

$$ {}^{C}I^{\nu } g(t)= \frac{1}{\Gamma (\nu )} \int _{0}^{t} (t-\upsilon )^{\nu -1}g(\upsilon ) \,d\upsilon . $$

### Definition 2

([24, 39])

For $g\in H^{1}(c,d)$ and $d>c$, the Caputo–Fabrizio derivative of fractional order $\nu \in (0,1)$ for *g* is given by

$$ {}^{CF}D^{\nu } g(t)=\frac{M(\nu )}{(1-\nu )} \int _{c}^{t} exp\biggl(\frac{-\nu }{1-\nu }(t- \upsilon )\biggr)g'(\upsilon )\,d\upsilon , $$

where $t\geq 0$, $M(\nu )$ is a normalization function that depends on *ν* and $M(0)=M(1)=1$. If $g \notin H^{1}(c,d)$ and $0<\nu <1$, this derivative for $g\in L^{1}(-\infty ,d)$ is given by

$$ {}^{CF}D^{\nu } g(t)=\frac{\nu M(\nu )}{(1-\nu )} \int _{- \infty }^{d} \bigl(g(t)-g(\upsilon )\bigr)exp \biggl(\frac{-\nu }{1-\nu }(t-\upsilon )\biggr)\,d \upsilon . $$

Also, the corresponding *CF* fractional integral is presented by

$$ {}^{CF}I^{\nu } g(t)=\frac{2(1-\nu )}{(2-\nu )M(\nu )} g(t)+ \frac{2\nu }{(2-\nu )M(\nu )} \int _{0}^{t} g(\upsilon )\,d \upsilon . $$

### Definition 3

([40])

Let $b>a$, $g\in H^{1}(a,b)$, and $0< \nu < 1$, then the fractional Atangana–Baleanu derivative in the Caputo sense is defined by

$$ {}^{ABC}D^{\nu }g(t)=\frac{B(\nu )}{(1-\nu )} \int _{a}^{t} g^{\prime }(s)E_{\nu } \biggl(\frac{-\nu }{1-\nu }(t-\upsilon )^{\nu }\biggr)\,d\upsilon , $$

where $B(\nu )$ denotes the normalization function satisfying $B(0)=B(1)=1$ and $E_{\nu }(\cdot)$ is a one-parameter Mittag-Leffler function. Also, the Atangana–Baleanu fractional integral is given as follows:

$$ {}^{ABC}I^{\nu }g(t)=\frac{1-\nu }{B(\nu )}g(t)+ \frac{\nu }{B(\nu )\Gamma (\nu )} \int _{a}^{t} g( \upsilon ) (t-\upsilon )^{\nu -1}\,d\upsilon . $$

The Laplace transform is one of the important tools in solving differential equations that are defined below for two kinds of fractional derivative.

### Definition 4

([38])

The Laplace transform of the Caputo fractional differential operator of order *ν* is given by

$$ L\bigl[{}^{C} D^{\nu }g(t)\bigr](s)=s^{\nu }L{g(t)}-\sum _{i=0}^{m-1}s^{\nu -i-1}g^{(i)}(0), \quad m-1 < \nu \leq m\in N, $$

which can also be obtained in the form

$$ L\bigl[{}^{C} D^{\nu }g(t)\bigr]= \frac{s^{m} L[g(t)]-s^{m-1}g(0)-s^{m-1}g^{\prime }(0)-\cdots-g^{(m-1)}}{s^{m-\nu }}. $$

## Model formulation

**Figure 1:**
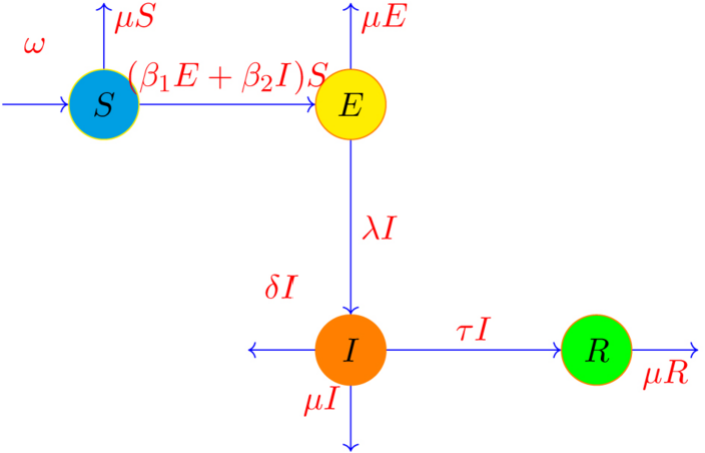
The diagram for the proposed model of COVID-19

In viral epidemic diseases, mathematical models are very important for predicting the transmission of the virus by considering its behavior in different regions for helping to manage the disease. Different mathematical models such as SIR, SEIR, SIRD, SEIRD, SIRS, SIRC, MSEIR, SEAIHRD, etc. are used to investigate the spread of diseases. According to the information published about COVID-19 by the World Health Organization, there are two types of people with the disease: one group has no symptoms and the other group has symptoms. Both groups transmit the disease to healthy people, and the sick people either recover or die. Of course, during this process, more groups can be considered, such as people who are hospitalized, but because we do not have accurate information about these groups for simulation, we decided to use the simple SEIR model. In this model, we divide people into four groups: susceptible people (S), exposed or asymptomatic infected people (E), symptomatic infected people (I), and recovered people (R) including improved people. The diagram for the dynamics of COVID-19 disease model is shown in Fig. 1.

Based on Fig. 1, we consider the SEIR model for COVID-19 as follows:

$$ \textstyle\begin{cases} \frac{dS}{dt}=\omega -(\beta _{1}E+\beta _{2}I)S-\mu S, \\ \frac{dE}{dt}=(\beta _{1}E+\beta _{2}I)S-(\lambda +\mu ) E , \\ \frac{dI}{dt}= \lambda E-(\tau +\mu +\delta ) I, \\ \frac{dR}{dt}=\tau I-\mu R, \end{cases} $$

where

- $\omega = n \times N$, *N* is the total number of individuals and *n* is the birth rate
- *μ*: the death rate of people,
- $\beta _{1}$: the transmission rate of infection from *E* to *S*,
- $\beta _{2}$: the transmission rate of infection from *I* to *S*,
- *λ*: the transmission rate of people from *E* to *I*,
- *δ*: the mortality rate due to the disease,
- *τ*: the rate of recovery of infected people,

with the initial conditions $S(0)=S_{0}>0$, $E(0)=E_{0}> 0$, $I(0)=I_{0} > 0$, $R(0)=R_{0}\geq 0$.

In this section, we moderate the system by substituting the time derivative with the Caputo fractional derivative. The ordinary derivative has an inverse second dimension $s^{-1}$ and the fractional derivative $D^{\nu }$ has a dimension of $s^{-\nu }$. To solve this problem, we use an auxiliary parameter *θ* that has a second dimension *s* and is called the cosmic time [41]. By the parameter, from a physical point of view, we will have $[\theta ^{\nu -1} {}^{C} {D}^{\nu }]=[\frac{d}{dt}]=s^{-1}$.

According to the explanation presented, the COVID-19 fractional model for $t > 0$ and $\nu \in (0,1)$ is given as follows:

1 $$ \textstyle\begin{cases} \theta ^{\nu -1} {}^{C} {D}^{\nu }_{t} S(t)=\omega -(\beta _{1}E(t)+\beta _{2}I(t))S(t)-\mu S(t), \\ \theta ^{\nu -1} {}^{C} {D}^{\nu }_{t}E(t)=(\beta _{1}E(t)+\beta _{2}I(t))S(t)-(\lambda +\mu ) E(t), \\ \theta ^{\nu -1} {}^{C} {D}^{\nu }_{t} I(t)=\lambda E(t)-(\tau +\mu +\delta ) I(t), \\ \theta ^{\nu -1} {}^{C} {D}^{\nu }_{t} R(t)=\tau I(t)-\mu R(t), \end{cases} $$

where the initial conditions are $S(0)=S_{0}>0$, $E(0)=E_{0}> 0$, $I(0)=I_{0} > 0$, $R(0)=R_{0}\geq 0$.

### Nonnegative solution

Consider $\Upsilon =\{(S,E,I,R)\in R^{+}_{4}: S+E+I+R\leq \frac{\omega }{\mu }\}$, we show that the closed set ϒ is the feasibility region of system (1).

#### Lemma 5

*The closed set* ϒ *is positively invariant with respect to fractional system* (1).

#### Proof

To obtain the fractional derivative of total population, we add all the relations in system (1). So

$$\begin{aligned} \theta ^{\nu -1} {}^{C} {D}^{\nu }_{t} N(t)&= \omega -\mu N(t)-\delta I(t) \\ &\leq \omega -\mu N(t), \end{aligned}$$

where $N(t)=S(t)+E(t)+I(t)+R(t)$. Using the Laplace transform and Theorem 7.2 (and Remark 7.1) in [42], we obtain

$$ N(t)\leq N(0)E_{\nu }\bigl(-\mu \theta ^{1-\nu }t^{\nu } \bigr)+ \int _{0}^{t} \omega \theta ^{1-\nu } \eta ^{\nu -1}E_{\nu ,\nu }\bigl(-\mu \theta ^{1- \nu }\eta ^{\nu }\bigr)\,d\eta , $$

where $N(0)$ is the initial population size. With some calculations, we get

$$\begin{aligned} N(t)&\leq N(0)E_{\nu }\bigl(-\mu \theta ^{1-\nu }t^{\nu } \bigr)+ \int _{0}^{t} \omega \theta ^{1-\nu } \eta ^{\nu -1} \sum_{i=0}^{\infty } \frac{(-1)^{i}\mu ^{i}\theta ^{i(1-\nu )}\eta ^{i\nu }}{\Gamma (i\nu +\nu )}\,d \eta \\ &=\frac{\omega \theta ^{1-\nu }}{\mu \theta ^{1-\nu }}+E_{\nu }\bigl(-\mu \theta ^{1-\nu }t^{\nu } \bigr) \biggl(N(0)- \frac{\omega \theta ^{1-\nu }}{\mu \theta ^{1-\nu }}\biggr) \\ &=\frac{\omega }{\mu }+E_{\nu }\bigl(-\mu \theta ^{1-\nu }t^{\nu } \bigr) \biggl(N(0)- \frac{\omega }{\mu }\biggr). \end{aligned}$$

Thus, if $N(0)\leq \frac{\omega }{\mu }$, then for $t>0$, $N(t)\leq \frac{\omega }{\mu }$. Consequently, the closed set ϒ is positively invariant with respect to fractional model (1). □

### Equilibrium points

To determine the equilibrium points of fractional-order system (1), we solve the following equations:

$$ {}^{C} {D}^{\nu }S(t)={}^{C} {D}^{\nu }E(t)={}^{C} {D}^{\nu }I(t)={}^{C} {D}^{\nu }R(t)=0. $$

By solving the algebraic equations, we obtain equilibrium points of system (1). The disease-free equilibrium point is obtained as $E_{0}=(\frac{\omega }{\mu },0,0,0,0,0)$. In addition, if $R_{0}>1$, then system (1) has a positive endemic equilibrium point $E_{1}=(S^{*},E^{*},I^{*},R^{*})$, so that

$$\begin{aligned}& S^{*}= \frac{(\lambda +\mu )(\tau +\mu +\delta )}{\beta _{1}(\tau +\mu +\delta )+\beta _{2}\lambda }, \\& E^{*}= \frac{\beta _{1}\omega (\tau +\mu +\delta )+\beta _{2}\lambda \omega -\mu (\lambda +\mu )(\tau +\mu +\delta )}{(\lambda +\mu )(\beta _{1}(\tau +\mu +\delta )+\beta _{2}\lambda )}, \\& I^{*}= \frac{\lambda (\beta _{1}\omega (\tau +\mu +\delta )+\beta _{2}\lambda \omega -\mu (\lambda +\mu )(\tau +\mu +\delta ))}{(\lambda +\mu )(\beta _{1}(\tau +\mu +\delta ) +\beta _{2}\lambda )(\tau +\mu +\delta )}, \\& R^{*}= \frac{\tau \lambda (\beta _{1}\omega (\tau +\mu +\delta )+\beta _{2}\lambda \omega -\mu (\lambda +\mu )(\tau +\mu +\delta ))}{(\lambda +\mu )(\beta _{1}(\tau +\mu +\delta )+\beta _{2}\lambda )(\tau +\mu +\delta )\mu }. \end{aligned}$$

Also, $R_{0}$ is the basic reproduction number and is obtained using the next generation method [43]. To find $R_{0}$, we first consider the system as follows:

$$ {}^{C} {D}^{\nu }\Psi (t)=F\bigl(\Psi (t)\bigr)-V\bigl(\Psi (t) \bigr), $$

where

$$ F\bigl(\Psi (t)\bigr)=\theta ^{1-\nu } \begin{bmatrix} (\beta _{1}E(t)+\beta _{2}I(t))S(t) \\ 0 \end{bmatrix} $$

and

$$ V\bigl(\Psi (t)\bigr)=\theta ^{1-\nu } \begin{bmatrix} (\lambda +\mu )E(t) \\ -\lambda E(t)+(\tau +\mu +\delta ) I(t) \end{bmatrix}. $$

At $E^{0}$, the Jacobian matrix for *F* and *V* is obtained as follows:

$$ J_{F}(E_{0})=\theta ^{1-\nu } \begin{bmatrix} \beta _{1}\frac{\omega }{\mu } & \beta _{2}\frac{\omega }{\mu } \\ 0& 0 \end{bmatrix} , \qquad J_{v}(E_{0})= \theta ^{1-\nu } \begin{bmatrix} \lambda +\mu & 0 \\ -\lambda & \tau +\mu +\delta \end{bmatrix} . $$

$FV^{-1}$ is the next generation matrix for system (1) and the basic reproduction number is obtained from $R_{0}=\rho (FV^{-1})$, so we get

$$ R_{0}= \frac{\beta _{1}\omega (\tau +\mu +\delta )+\beta _{2}\omega \lambda }{\mu (\lambda +\mu )(\tau +\mu +\delta )}. $$

This basic reproduction number $R_{0}$ is an epidemiologic metric used to describe the contagiousness or transmissibility of infectious agents.

### $R_{0}$ sensitivity analysis

To check the $R_{0}$ sensitivity, we calculate its derivatives as follows:

$$\begin{aligned}& \frac{\partial R_{0}}{\partial \beta _{1}}= \frac{\omega }{\mu (\lambda +\mu )}, \\& \frac{\partial R_{0}}{\partial \beta _{2}}= \frac{\omega \lambda }{\mu (\lambda +\mu )(\tau +\mu +\delta )}, \\& \frac{\partial R_{0}}{\partial \omega }= \frac{\beta _{1}(\tau +\mu +\delta )+\beta _{2}\lambda }{\mu (\lambda +\mu )(\tau +\mu +\delta )}, \\& \frac{\partial R_{0}}{\partial \lambda }= \frac{\beta _{2}\omega (2\lambda +\mu )-\beta _{1}\omega (\tau +\mu +\delta )}{\mu (\lambda +\mu )^{2}(\tau +\mu +\delta )}, \\& \frac{\partial R_{0}}{\partial \tau }= \frac{\beta _{1}\omega (\tau +\mu +\delta )^{2}-\beta _{1}\omega (\tau +\mu +\delta )+\beta _{2}\omega \lambda }{\mu (\lambda +\mu )(\tau +\mu +\delta )^{2}}, \\& \frac{\partial R_{0}}{\partial \delta }= \frac{\beta _{1}\omega (\tau +\mu +\delta )^{2}-\beta _{1}\omega (\tau +\mu +\delta )+\beta _{2}\omega \lambda }{\mu (\lambda +\mu )(\tau +\mu +\delta )^{2}}, \\& \begin{aligned} \frac{\partial R_{0}}{\partial \mu }&= \frac{\beta _{1}\omega \mu -\beta _{1}\omega (\tau +\mu +\delta )-\beta _{2}\omega \lambda }{\mu ^{2}(\lambda +\mu )(\tau +\mu +\delta )} \\ &\quad {}- \frac{\beta _{1}\omega (\tau +\mu +\delta )+\beta _{2}\omega \lambda }{\mu (\lambda +\mu )^{2}(\tau +\mu +\delta )}- \frac{\beta _{1}\omega (\tau +\mu +\delta ) +\beta _{2}\omega \lambda }{\mu (\lambda +\mu )(\tau +\mu +\delta )^{2}} . \end{aligned} \end{aligned}$$

Because all the parameters are positive, so $\frac{\partial R_{0}}{\partial \beta _{1}}> 0$, $\frac{\partial R_{0}}{\partial \beta _{2}}> 0$, $\frac{\partial R_{0}}{\partial \omega }> 0$. Thus $R_{0}$ is increasing with $\beta _{1}$, $\beta _{2}$, *ω*, but we cannot say anything about other parameters here.

## Stability of equilibrium points

In this section we investigate the stability of equilibrium points. The Jacobian matrix of system (1) is obtained as follows:

$$ J=\theta ^{1-\nu } \begin{bmatrix} -(\beta _{1}E+\beta _{2}I)-\mu & -\beta _{1}S & -\beta _{2}S & 0 \\ \beta _{1}E+\beta _{2}I & \beta _{1}S-(\lambda +\mu ) & \beta _{2}S & 0 \\ 0 & \lambda & -(\tau +\mu +\delta ) & 0 \\ 0 & 0 & \tau & -\mu \end{bmatrix}. $$

So, the Jacobian matrix of system at $E_{0}$ is

$$ J(E_{0})=\theta ^{1-\nu } \begin{bmatrix} -\mu & -\beta _{1}\frac{\omega }{\mu } & -\beta _{2}\frac{\omega }{\mu } & 0 \\ 0 & \beta _{1}\frac{\omega }{\mu } -(\lambda +\mu ) & \beta _{2} \frac{\omega }{\mu } & 0 \\ 0 & \lambda & -(\tau +\mu +\delta ) & 0 \\ 0 & 0 & \tau & -\mu \end{bmatrix}. $$

### Theorem 6

*The equilibrium point* $E_{0}$*of system* (1) *is locally asymptotically stable if* $R_{0}< 1$*and* $E_{0}$*is unstable if* $R_{0}>1$.

### Proof

The characteristic equation of the Jacobian matrix at the disease-free equilibrium point $J(E_{0})$ is $det(J(E_{0})-kI)=0$. Then we obtain

$$ -\theta ^{1-\nu }(k+\mu )^{2}\bigl(k^{2}+Ak+B \bigr)=0, $$

where $A=- \frac{\beta _{1}\omega -\mu (\lambda +\mu )-\mu (\tau +\mu +\delta )}{\mu }$ and $B=- \frac{\beta _{1}\omega (\tau +\mu +\delta )-\mu (\lambda +\mu )(\tau +\mu +\delta )+\lambda \beta _{2}\omega }{\mu }$. The eigenvalues of the characteristic equation are $k=-\mu $ and the roots of the equation

$$ k^{2}+Ak+B=0. $$

If $R_{0}<1$, since all of the parameters are positive, then

$$\begin{aligned}& \beta _{1}\omega (\tau +\mu +\delta )+\beta _{2}\omega \lambda < \mu ( \lambda +\mu ) (\tau +\mu +\delta ), \\& \frac{\beta _{1}\omega (\tau +\mu +\delta )+\beta _{2}\omega \lambda -\mu (\lambda +\mu )(\tau +\mu +\delta )}{\mu }< 0 \quad \Rightarrow \quad A > 0. \end{aligned}$$

Also, from $R_{0}< 1$ we have

$$ \begin{aligned} \beta _{1}\omega -\mu (\lambda +\mu )< \frac{-\beta _{2}\omega \lambda }{\tau +\mu +\delta } < 0 &\quad \Rightarrow\quad \frac{\beta _{1}\omega -\mu (\lambda +\mu )-\mu (\tau +\mu +\delta )}{\mu }< 0 \\ &\quad \Rightarrow\quad B>0{.} \end{aligned} $$

Applying the Routh–Hurwitz criterion, $E_{0}$ is locally asymptotically stable. If $R_{0}> 1$, then $B<0$, and there is one positive real root for Eq.(ref2), then $E_{0}$ will be unstable. □

The Jacobian matrix of system (1) at the endemic equilibrium point is

$$ J(E_{1})=\theta ^{1-\nu } \begin{bmatrix} -(\beta _{1}E^{*}+\beta _{2}I^{*})-\mu & -\beta _{1}S^{*} & -\beta _{2}S^{*} & 0 \\ \beta _{1}E^{*}+\beta _{2}I^{*} & \beta _{1}S^{*}-(\lambda +\mu ) & \beta _{2}S^{*} & 0 \\ 0 & \lambda & -(\tau +\mu +\delta ) & 0 \\ 0 & 0 & \tau & -\mu \end{bmatrix}. $$

The characteristic equation of matrix $J(E_{1})$ is obtained as follows:

$$ \theta ^{1-\nu }(k+\mu ) \bigl(k+(\mu +\delta +\tau )\bigr) \bigl(k^{2} -A_{1}k+B_{1}\bigr)=0, $$

where

$$\begin{aligned}& A_{1}=\beta _{1}S^{*}-\lambda -2\mu + \frac{\beta _{2}S^{*}\lambda }{\mu +\tau +\delta }, \\& B_{1}=\bigl(\mu +\beta _{1}E^{*}+\beta _{2}I^{*}\bigr) (\lambda +\mu )+\biggl( \frac{(\beta _{1}S^{*}-\lambda -\mu )(\mu +\tau +\delta )+\beta _{2}S^{*}\lambda }{\mu +\tau +\delta } \biggr). \end{aligned}$$

The eigenvalues of the characteristic equation are $k_{1}=-\mu $, $k_{2}=-(\mu +\delta +\tau )$ and the roots of the equation

2 $$ k^{2} -A_{1}k+B_{1}=0. $$

Since $k_{1}$, $k_{2}$ are negative, so $E_{1}$ is locally asymptotically stable when two roots of Eq. (2) are negative, so it is enough to have $B_{1}> 0$ and $A_{1}< 0$.

## Existence and uniqueness of solution

In this section we show that the system has a unique solution. First, we write system (1) as follows:

$$ \textstyle\begin{cases} \theta ^{\nu -1} {}^{C} {D}^{\nu }_{t} S(t)=Q_{1}(t,S(t)), \\ \theta ^{\nu -1} {}^{C} {D}^{\nu }_{t} E(t)=Q_{2}(t,E(t)), \\ \theta ^{\nu -1} {}^{C} {D}^{\nu }_{t} I(t)=Q_{3}(t,I(t)), \\ \theta ^{\nu -1} {}^{C} {D}^{\nu }_{t} R(t)=Q_{4}(t,R(t)). \end{cases} $$

By taking integral form on both sides of the above equations, we get

3 $$ \textstyle\begin{cases} S(t)-S(0) =\frac{\theta ^{1-\nu }}{\Gamma (\nu )} \int _{0}^{t} Q_{1}(\tau ,S)(t-\tau )^{\nu -1}\,d\tau , \\ E(t)-E(0) =\frac{\theta ^{1-\nu }}{\Gamma (\nu )} \int _{0}^{t} Q_{2}(\tau ,E)(t-\tau )^{\nu -1}\,d\tau , \\ I(t)-I(0) =\frac{\theta ^{1-\nu }}{\Gamma (\nu )} \int _{0}^{t} Q_{3}(\tau ,I)(t-\tau )^{\nu -1}\,d\tau , \\ A(t)-A(0) =\frac{\theta ^{1-\nu }}{\Gamma (\nu )} \int _{0}^{t} Q_{4}(\tau ,A)(t-\tau )^{\nu -1}\,d\tau . \end{cases} $$

We show that the kernels $Q_{i},i=1,2,3,4$, satisfy the Lipschitz condition and contraction.

### Theorem 7

*The kernel* $Q_{1}$*satisfies the Lipschitz condition and contraction if the following inequality holds*:

$$ 0\leq \beta _{1} d_{2}+\beta _{2}d_{3}+ \mu < 1. $$

### Proof

For *S* and $S_{1}$ we have

$$\begin{aligned} \bigl\Vert Q_{1}(t,S)-Q_{1}(t,S_{1}) \bigr\Vert &= \bigl\Vert -\bigl(\beta _{1}E(t)+ \beta _{2}I(t) \bigr) \bigl(S(t)-S_{1}(t)\bigr)-\mu \bigl(S(t)-S_{1}(t)\bigr) \bigr\Vert \\ &\leq \bigl\Vert \beta _{1}E(t)+\beta _{2}I(t) \bigr\Vert \bigl\Vert S(t)-S_{1}(t) \bigr\Vert +\mu \bigl\Vert S(t)-S_{1}(t) \bigr\Vert \\ &\leq \bigl(\beta _{1} \bigl\Vert E(t) \bigr\Vert +\beta _{2}\|I(t)\bigr)\|+\mu ) \bigl\Vert S(t)-S_{1}(t) \bigr\Vert \\ &\leq (\beta _{1}d_{2}+\beta _{2}d_{3}+ \mu ) \bigl\Vert S(t)-S_{1}(t) \bigr\Vert . \end{aligned}$$

Suppose that $h_{1}=\beta _{1}d_{2}+\beta _{2}d_{3}+\mu $, where $\| E(t)\| \leq d_{2}$, $\| I(t)\| \leq d_{3}$ are bounded functions. So

4 $$ \bigl\Vert Q_{1}(t,S)-Q_{1}(t,S_{1}) \bigr\Vert \leq h_{1} \bigl\Vert \bigl(S(t)-S_{1}(t) \bigr) \bigr\Vert . $$

Thus, for $Q_{1}$ the Lipschitz condition is obtained, and if $0\leq \beta _{1}d_{2}+\beta _{2}d_{3}+\mu < 1 $, then $Q_{1}$ is a contraction. □

In the same way, we can prove that $Q_{j},j=2,3,4$, satisfy the Lipschitz condition as follows:

$$ \textstyle\begin{cases} \Vert Q_{2}(t,E)-Q_{2}(t,E_{1}) \Vert \leq h_{2} \Vert (E(t)-E_{1}(t)) \Vert , \\ \Vert Q_{3}(t,I)-Q_{3}(t,I_{1}) \Vert \leq h_{3} \Vert (I(t)-I_{1}(t)) \Vert , \\ \Vert Q_{4}(t,R)-Q_{4}(t,R_{1}) \Vert \leq h_{4} \Vert (R(t)-R_{1}(t)) \Vert , \end{cases} $$

where $\| S(t)\| \leq d_{1}$, and $h_{2}=\beta _{1}d_{1}+\lambda +\mu $, $h_{3}=\tau +\mu +\delta $, $h_{4}=\mu $ are bounded functions. If for $j=2,3,4$ we have $0\leq h_{j}<1$, then $Q_{j}$ are contractions for $j=2,3,4$. According to system (3), consider the following recursive forms:

$$\begin{aligned}& \psi _{1n}(t)=S_{n}(t)-S_{n-1}(t)= \frac{\theta ^{1-\nu }}{\Gamma (\nu )} \int _{0}^{t} \bigl(Q_{1}( \tau ,S_{n-1})-Q_{1}(\tau ,S_{n-2})\bigr) (t-\tau )^{\nu -1}\,d\tau , \\& \psi _{2n}(t)=E_{n}(t)-E_{n-1}(t)= \frac{\theta ^{1-\nu }}{\Gamma (\nu )} \int _{0}^{t} \bigl(Q_{2}( \tau ,E_{n-1})-Q_{2}(\tau ,E_{n-2})\bigr) (t-\tau )^{\nu -1}\,d\tau , \\& \psi _{3n}(t)=I_{n}(t)-I_{n-1}(t)= \frac{\theta ^{1-\nu }}{\Gamma (\nu )} \int _{0}^{t} \bigl(Q_{3}( \tau ,I_{n-1})-Q_{3}(\tau ,I_{n-2})\bigr) (t-\tau )^{\nu -1}\,d\tau , \\& \psi _{4n}(t)=R_{n}(t)-R_{n-1}(t)= \frac{\theta ^{1-\nu }}{\Gamma (\nu )} \int _{0}^{t} \bigl(Q_{4}( \tau ,R_{n-1})-Q_{4}(\tau ,R_{n-2})\bigr) (t-\tau )^{\nu -1}\,d\tau , \end{aligned}$$

with the initial conditions $S_{0}(t)=S(0)$, $E_{0}(t)=E(0)$, $I_{0}(t)=I(0)$, and $R_{0}(t)=R(0)$. We take the norm of the first equation in the above system, then

$$\begin{aligned} \bigl\Vert \psi _{1n}(t) \bigr\Vert &= \bigl\Vert S_{n}(t)-S_{n-1}(t) \bigr\Vert \\ &= \biggl\Vert \frac{\theta ^{1-\nu }}{\Gamma (\nu )} \int _{0}^{t} \bigl(Q_{1}( \tau ,S_{n-1})-Q_{1}(\tau ,S_{n-2})\bigr) (t-\tau )^{\nu -1}\,d\tau \biggr\Vert \\ &\leq \frac{\theta ^{1-\nu }}{\Gamma (\nu )} \int _{0}^{t} \bigl\| Q_{1}(\tau ,S_{n-1})-Q_{1}(\tau ,S_{n-2})) (t-\tau )^{\nu -1}\bigr\| \,d \tau , \end{aligned}$$

with Lipschitz condition (4), we have

5 $$ \bigl\Vert \psi _{1n}(t) \bigr\Vert \leq \frac{\theta ^{1-\nu }}{\Gamma (\nu )} h_{1} \int _{0}^{t} \bigl\Vert \psi _{1(n-1)}(\tau ) \bigr\Vert \,d\tau . $$

In a similar way, we obtain

6 $$\begin{aligned}& \bigl\Vert \psi _{2n}(t) \bigr\Vert \leq \frac{\theta ^{1-\nu }}{\Gamma (\nu )} h_{2} \int _{0}^{t} \bigl\Vert \psi _{2(n-1)}(\tau ) \bigr\Vert \,d\tau , \\& \bigl\Vert \psi _{3n}(t) \bigr\Vert \leq \frac{\theta ^{1-\nu }}{\Gamma (\nu )} h_{3} \int _{0}^{t} \bigl\Vert \psi _{3(n-1)}(\tau ) \bigr\Vert \,d\tau , \\& \bigl\Vert \psi _{4n}(t) \bigr\Vert \leq \frac{\theta ^{1-\nu }}{\Gamma (\nu )} h_{4} \int _{0}^{t} \bigl\Vert \psi _{4(n-1)}(\tau ) \bigr\Vert \,d\tau . \end{aligned}$$

Thus, we can write that

$$ S_{n}(t)=\sum_{i=1}^{n}\psi _{1i}(t), E_{n}(t)=\sum_{i=1}^{n} \psi _{2i}(t), I_{n}(t)=\sum_{i=1}^{n} \psi _{3i}(t), R_{n}(t)=\sum_{i=1}^{n} \psi _{4i}(t). $$

In the next theorem, we prove the existence of a solution.

### Theorem 8

*A system of solutions given by the fractional COVID*-19 *SEIR model* (1) *exists if there exists* $t_{1}$*such that*

$$ \frac{\theta ^{1-\nu }}{\Gamma (\nu )}t_{1}h_{j}< 1. $$

### Proof

From the recursive technique and Eq. (5) and Eq. (6) we conclude that

$$\begin{aligned}& \bigl\Vert \psi _{1n}(t) \bigr\Vert \leq \bigl\Vert S_{n}(0) \bigr\Vert \biggl[ \frac{\theta ^{1-\nu }}{\Gamma (\nu )}h_{1}t \biggr]^{n}, \\& \bigl\Vert \psi _{2n}(t) \bigr\Vert \leq \bigl\Vert E_{n}(0) \bigr\Vert \biggl[ \frac{\theta ^{1-\nu }}{\Gamma (\nu )}h_{2}t \biggr]^{n}, \\& \bigl\Vert \psi _{3n}(t) \bigr\Vert \leq \bigl\Vert I_{n}(0) \bigr\Vert \biggl[ \frac{\theta ^{1-\nu }}{\Gamma (\nu )}h_{3}t \biggr]^{n}, \\& \bigl\Vert \psi _{4n}(t) \bigr\Vert \leq \bigl\Vert R_{n}(0) \bigr\Vert \biggl[ \frac{\theta ^{1-\nu }}{\Gamma (\nu )}h_{4}t \biggr]^{n}. \end{aligned}$$

Thus, the system has a solution and also it is continuous. Now we show that the above functions construct solution for model (3). We assume that

$$\begin{aligned}& S(t)-S(0)=S_{n}(t)-\mathrm{B}_{1n}(t), \\& E(t)-E(0)=E_{n}(t)-\mathrm{B}_{2n}(t), \\& I(t)-I(0)=I_{n}(t)-\mathrm{B}_{3n}(t), \\& R(t)-R(0)=R_{n}(t)-\mathrm{B}_{4n}(t). \end{aligned}$$

So

$$\begin{aligned} \bigl\Vert \mathrm{B}_{1n}(t) \bigr\Vert &= \biggl\Vert \frac{\theta ^{1-\nu }}{\Gamma (\nu )} \int _{0}^{t} \bigl(Q_{1}(\tau ,S)-Q_{1}(\tau ,S_{n-1})\bigr)\,d \tau \biggr\Vert \\ &\leq \frac{\theta ^{1-\nu }}{\Gamma (\nu )} \int _{0}^{t} \bigl\Vert Q_{1}(\tau ,S)-Q_{1}(\tau ,S_{n-1}) \bigr\Vert \,d\tau \\ &\leq \frac{\theta ^{1-\nu }}{\Gamma (\nu )}h_{1} \Vert S-S_{n-1} \Vert t. \end{aligned}$$

By repeating the method, we obtain

$$ \bigl\Vert \mathrm{B}_{1n}(t) \bigr\Vert \leq \biggl[ \frac{\theta ^{1-\nu }}{\Gamma (\nu )} t\biggr]^{n+1} h_{1}^{n+1}k. $$

At $t_{1}$, we get

$$ \bigl\Vert \mathrm{B}_{1n}(t) \bigr\Vert \leq \biggl[ \frac{\theta ^{1-\nu }}{\Gamma (\nu )} t_{1}\biggr]^{n+1} h_{1}^{n+1}k. $$

Taking limit on the recent equation as *n* approaches ∞, we obtain $\| \mathrm{B}_{1n}(t)\|\rightarrow 0$. In the same way, we can show that $\| \mathrm{B}_{jn}(t)\|\rightarrow 0$, $j=2,3,4$. This completes the proof. □

To show the uniqueness of the solution, we suppose that the system has another solution such as $S_{1}(t)$, $E_{1}(t)$, $I_{1}(t)$, and $R_{1}(t)$, then we have

$$ S(t)-S_{1}(t)=\frac{\theta ^{1-\nu }}{\Gamma (\nu )} \int _{0}^{t} \bigl(Q_{1}(\tau ,S)-Q_{1}(\tau ,S_{1})\bigr)\,d \tau . $$

We take norm from this equation

$$ \bigl\Vert S(t)-S_{1}(t) \bigr\Vert =\frac{\theta ^{1-\nu }}{\Gamma (\nu )} \int _{0}^{t} \bigl\Vert Q_{1}(\tau ,S)-Q_{1}(\tau ,S_{1}) \bigr\Vert \,d \tau . $$

It follows from Lipschitz condition (4) that

$$ \bigl\Vert S(t)-S_{1}(t) \bigr\Vert \leq \frac{\theta ^{1-\nu }}{\Gamma (\nu )}h_{1} t \bigl\Vert S(t)-S_{1}(t) \bigr\Vert . $$

Thus

7 $$ \bigl\Vert S(t)-S_{1}(t) \bigr\Vert \biggl(1- \frac{\theta ^{1-\nu }}{\Gamma (\nu )}h_{1}t\biggr) \leq 0. $$

### Theorem 9

*The solution of COVID*-19 *SEIR model* (1) *is unique if the following condition holds*:

$$ 1-\frac{\theta ^{1-\nu }}{\Gamma (\nu )}h_{1}t > 0. $$

### Proof

Suppose that condition (7) holds

$$ \bigl\Vert S(t)-S_{1}(t) \bigr\Vert \biggl(1- \frac{\theta ^{1-\nu }}{\Gamma (\nu )} h_{1}t\biggr) \leq 0. $$

Then $\|S(t)-S_{1}(t)\|=0$. So, we obtain $S(t)=S_{1}(t)$. Similarly, we can show the same equality for *E*, *I*, *R*. □

## Numerical results

Using the fractional Euler method for Caputo derivative, we present approximate solutions for the fractional-order COVID-19 SEIR model [44]. We present simulations to predict the COVID-19 transmission in the world.

### Numerical method

We consider system (1) in a compact form as follows:

8 $$ \theta ^{\nu -1} {}^{C} {D}^{\nu }_{t} w(t)=g\bigl(t,w(t)\bigr), \quad w(0)=w_{0}, 0\leq t \leq T< \infty , $$

where $w=(S,E,I,R)\in R^{4}_{+}$, $w_{0}=(S_{0},E_{0},I_{0},R_{0})$ is the initial vector, and $g(t)\in R$ is a continuous vector function satisfying Lipschitz condition

$$ \bigl\Vert g\bigl(w_{1}(t)\bigr)-g\bigl(w_{2}(t)\bigr) \bigr\Vert \leq k \bigl\Vert w_{1}(t)-w_{2}(t) \bigr\Vert , \quad k> 0. $$

Applying a fractional integral operator corresponding to the Caputo derivative to equation (8), we obtain

$$ w(t)=\theta ^{1-\nu }\bigl[w_{0}+I^{\nu } g\bigl(w(t) \bigr)\bigr], \quad 0\leq t \leq T< \infty . $$

Set $h=\frac{T-0}{N}$ and $t_{n}=nh$, where $t\in [0,T]$ and *N* is a natural number and $n=0,1,2,\ldots,N$. Let $w_{n}$ be the approximation of $w(t)$ at $t=t_{n}$. Using the fractional Euler method [44], we get

$$ w_{n+1}=\theta ^{1-\nu }\Biggl[w_{0}+ \frac{h^{\nu }}{\Gamma (\nu +1)}\sum_{j=0}^{n}u_{n+1,j}g(t_{j},w_{j}) \Biggr], \quad j=0,1,2,\ldots,N-1, $$

where

$$ u_{n+1,j}=(n+1-j)^{\nu }-(n-j)^{\nu }, \quad j=0,1,2, \ldots,n. $$

The stability analysis of the obtained scheme has been proved in Theorem (3.1) in [44].

Thus, the solution of system (1) is written as follows:

$$\begin{aligned}& S_{n+1}=\theta ^{1-\nu }\Biggl[S_{0}+ \frac{h^{\nu }}{\Gamma (\nu +1)}\sum_{j=0}^{n}u_{n+1,j}f_{1}(t_{j},w_{j}) \Biggr], \\& E_{n+1}=\theta ^{1-\nu }\Biggl[E_{0}+ \frac{h^{\nu }}{\Gamma (\nu +1)}\sum_{j=0}^{n}u_{n+1,j}f_{2}(t_{j},w_{j}) \Biggr], \\& I_{n+1}=\theta ^{1-\nu }\Biggl[I_{0}+ \frac{h^{\nu }}{\Gamma (\nu +1)}\sum_{j=0}^{n}u_{n+1,j}f_{3}(t_{j},w_{j}) \Biggr], \\& R_{n+1}=\theta ^{1-\nu }\Biggl[R_{0}+ \frac{h^{\nu }}{\Gamma (\nu +1)}\sum_{j=0}^{n}u_{n+1,j}f_{4}(t_{j},w_{j}) \Biggr], \end{aligned}$$

where $u_{n+1,j}=(n+1-j)^{\nu }-(n-i)^{\nu }$, $f_{1}(t,w(t))=\omega -(\beta _{1}E(t)+\beta _{2}I(t))S(t)-\mu S(t)$, $f_{2}(t,w(t))=( \beta _{1}E(t)+\beta _{2}I(t))S(t)-(\lambda +\mu ) E(t)$, $f_{3}(t,w(t))=\lambda E(t)-(\tau +\mu +\delta ) I(t)$, $f_{4}(t,w(t))=\tau I(t)-\mu R(t)$.

### Simulation

*Case I: The world*

**Figure 2:**
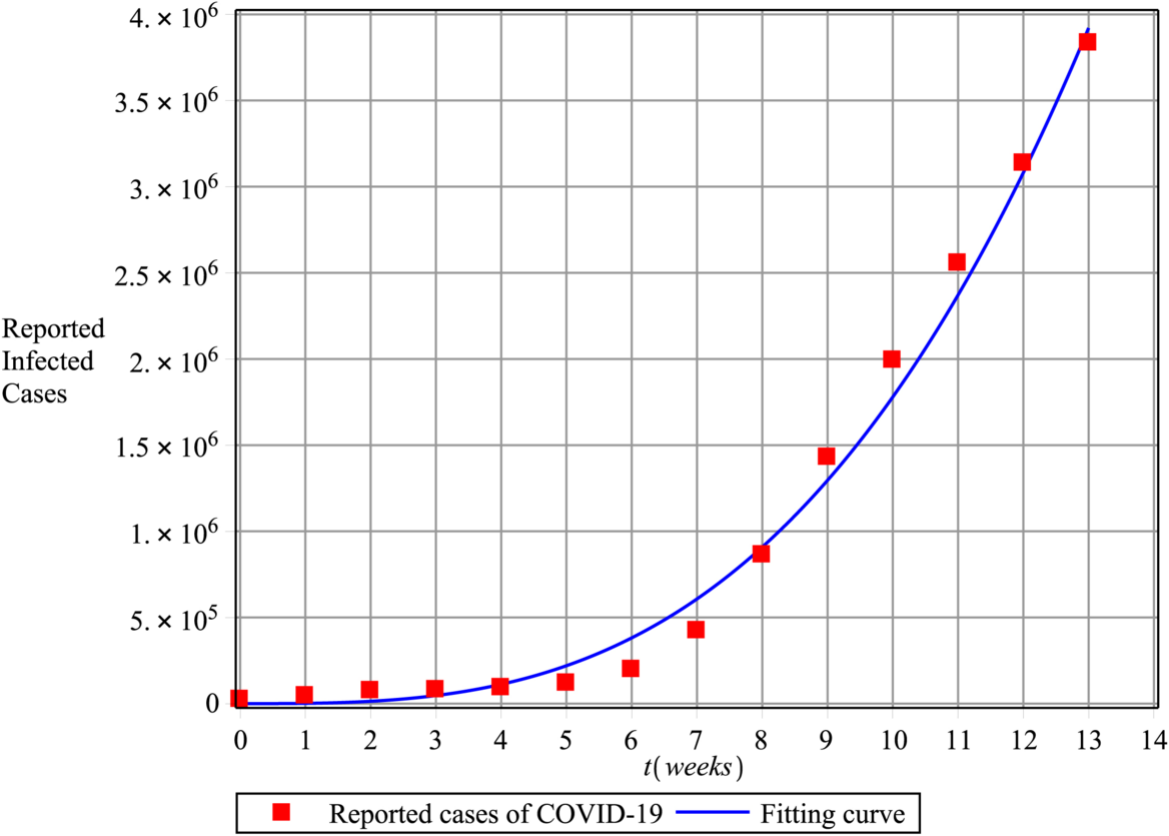
The fitted curve and the reported total cases of COVID-19 in the world from 4 February to 12 May

To provide a numerical simulation, we must first determine the value of the parameters. The current birth rate for the world in 2020 is 18.077 births per 1000 people, and the death rate is 7.612 per 1000 people [45]. The world’s population on 4 February was $N=7610105452$, so $\omega =\frac{n\times N}{365}=391347.066$ and $\mu =\frac{7.612}{365\times 1000}=2.08547\times 10^{-5}$, and we choose $\theta =0.99$. Since $N(0)=S(0)+E(0)+I(0)+R(0)$ and on 4 February we have $I(0)=24545$, $R(0)=907$ [46], then we assume $E(0)=80000$ and $S(0)=7610026000$. Also, according to the WHO report, the COVID-19 mortality rate is $\delta =3.4\times 10^{-2}$ [46]. To estimate other parameters, we use the fitting curve technique with the real data reported for COVID-19. The fitted curve and the reported cumulative number of COVID-19 in the world from 4 February to 12 May 2020 are plotted in Fig. 2, so that every part is a week. Using this method, we obtain the parameters as follows: $\beta _{1}=2\times 10^{-11}$, $\beta _{2}=2.2\times 10^{-9}$, $\lambda =2.35 \times 10^{-5}$, $\tau = 0.03$.

Table 1 compares the absolute and relative errors for I(t) concerning the fractional- and integer-order models with respect to the reported cases of infected people. From this table, you can observe that the Caputo model with $\nu = 0.97$ provides more realistic results than the classic model with integer-order derivatives.

**Table 1:** The absolute and relative errors for $I(t)$

Model	*ν*	Absolute error	Relative error
Fractional	0.97	7.23451	0.0312
Integer	−	9.04562	0.0394

**Figure 3:**
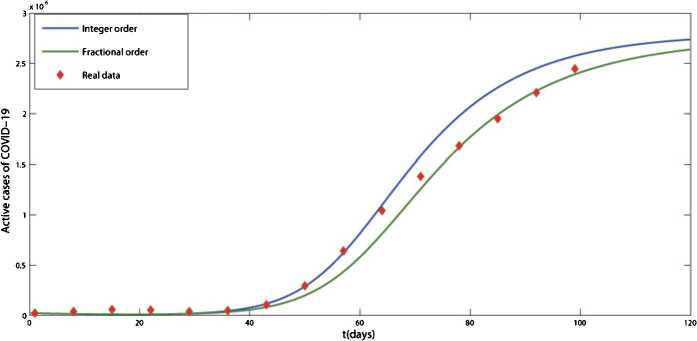
Comparison between the results of the integer-order derivative $\nu =1$ and the fractional-order derivative $\nu =0.97$ with real data

A comparison between the noninteger-order model with $\nu = 0.97$ and the integer-order one with $\nu =1$ and the real data for the infected cases of the COVID-19 from 4 February to 12 May is also given in Fig. 3. The obtained results show that the answer of the fractional-order model corresponds well with the real data and together with the results of Table 1 shows the advantage of using the fractional-order derivative instead of the correct order one.

**Figure 4:**
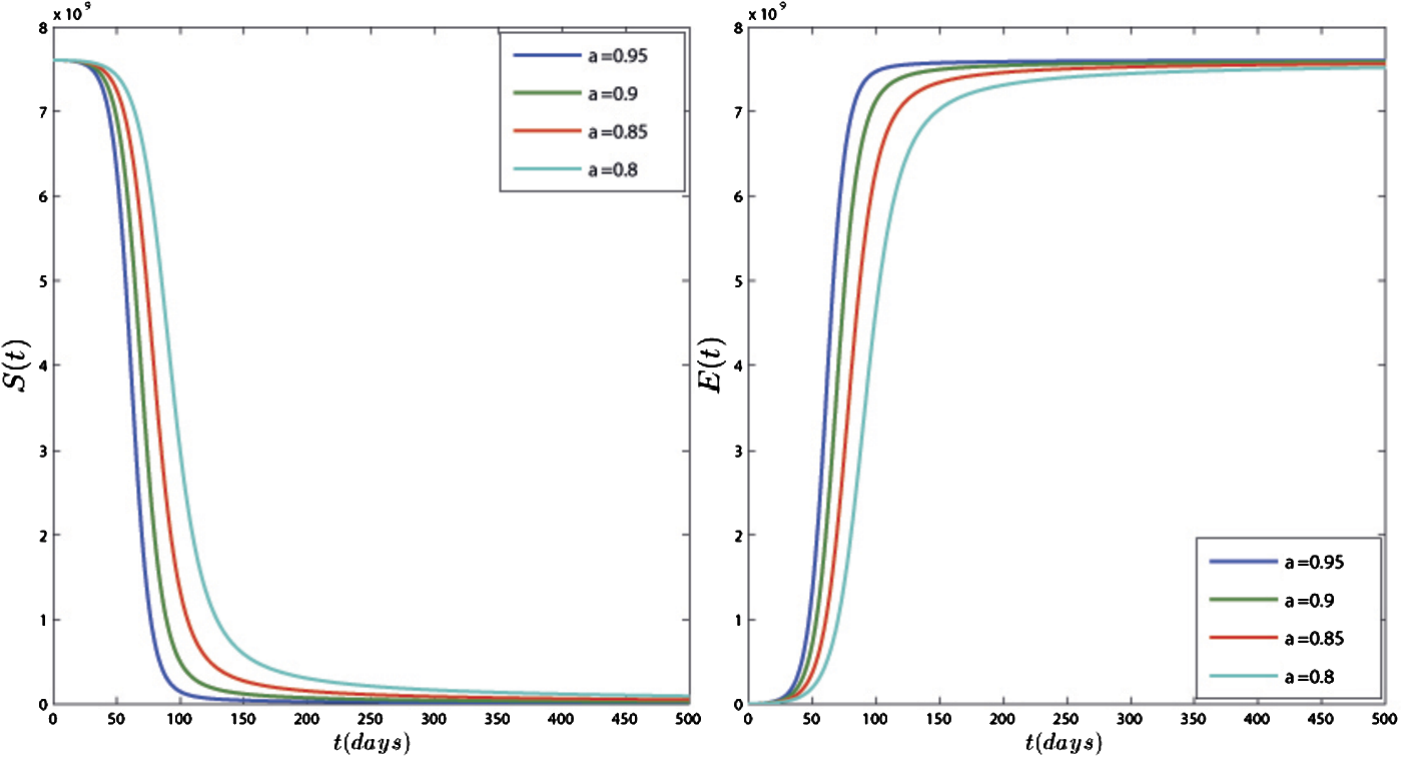
Dynamics of S(t) and E(t) for different values of $\nu =0.95, 0.9, 0.85, 0.8$

**Figure 5:**
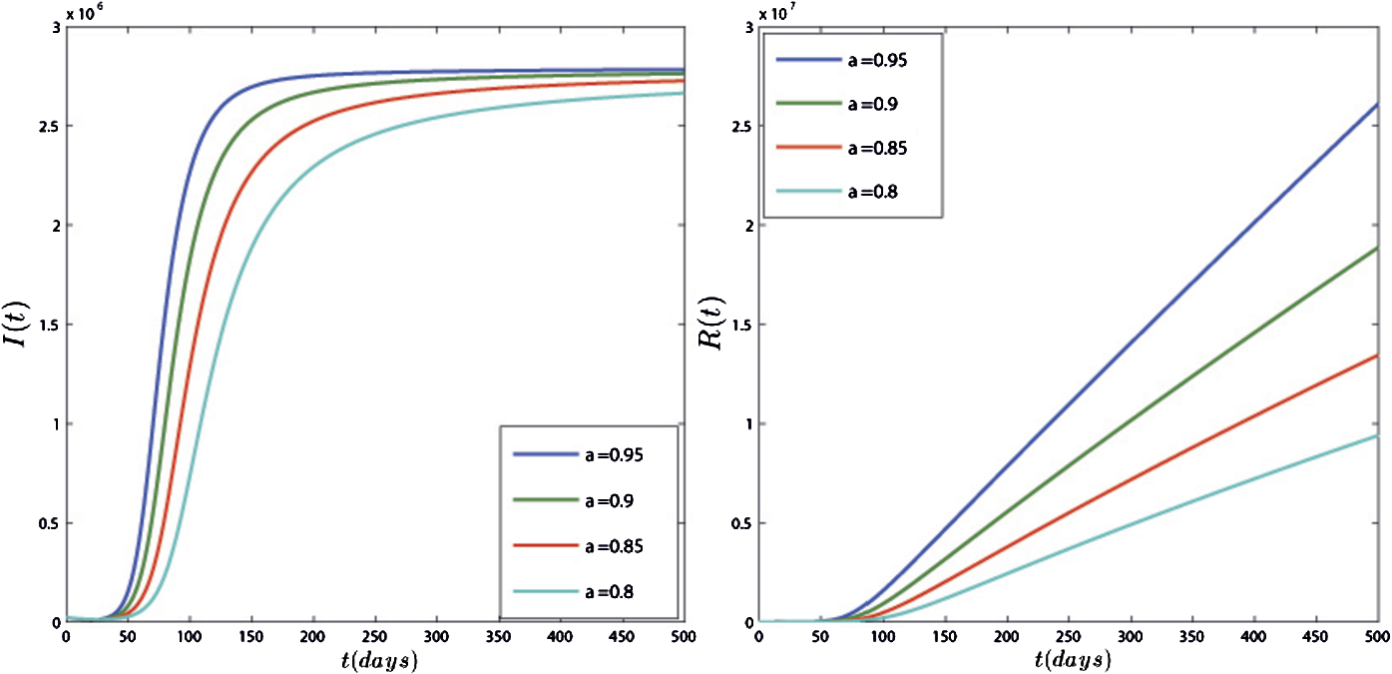
Dynamics of I(t) and R(t) for different values of $\nu =0.95, 0.9, 0.85, 0.8$

In Figs. 4 and 5, we have plotted the results of model (1) for different values of *ν*. In this simulation, the equilibrium point is

$$ E_{1}=\bigl(S^{*},E^{*},I^{*},R^{*} \bigr)=\bigl(2.5\times 10^{6}, 7.52\times 10^{9}, 2.76 \times 10^{6}, 2.9\times 10^{9}\bigr). $$

Figures 4 and 5 show that the results of the model converge to their equilibrium point for different orders of derivation, and the results of all orders are stable at the equilibrium points. These figures show that the obtained plots for different values of *ν* are different in quantity but they have the same behavior. Also, Fig. 5 shows that approximately 50 days after 4 February 2020 the number of active cases ceases to increase and becomes stable in $2.76\times 10^{6}$.

*Case II: Iran*

**Figure 6:**
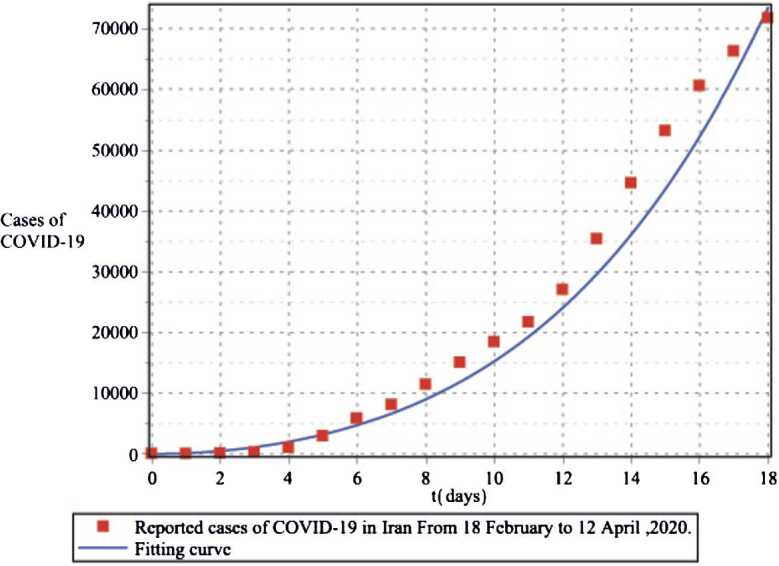
The fitted curve and the reported cases of COVID-19 in the Iran from 18 February to 12 April 2020

**Figure 7:**
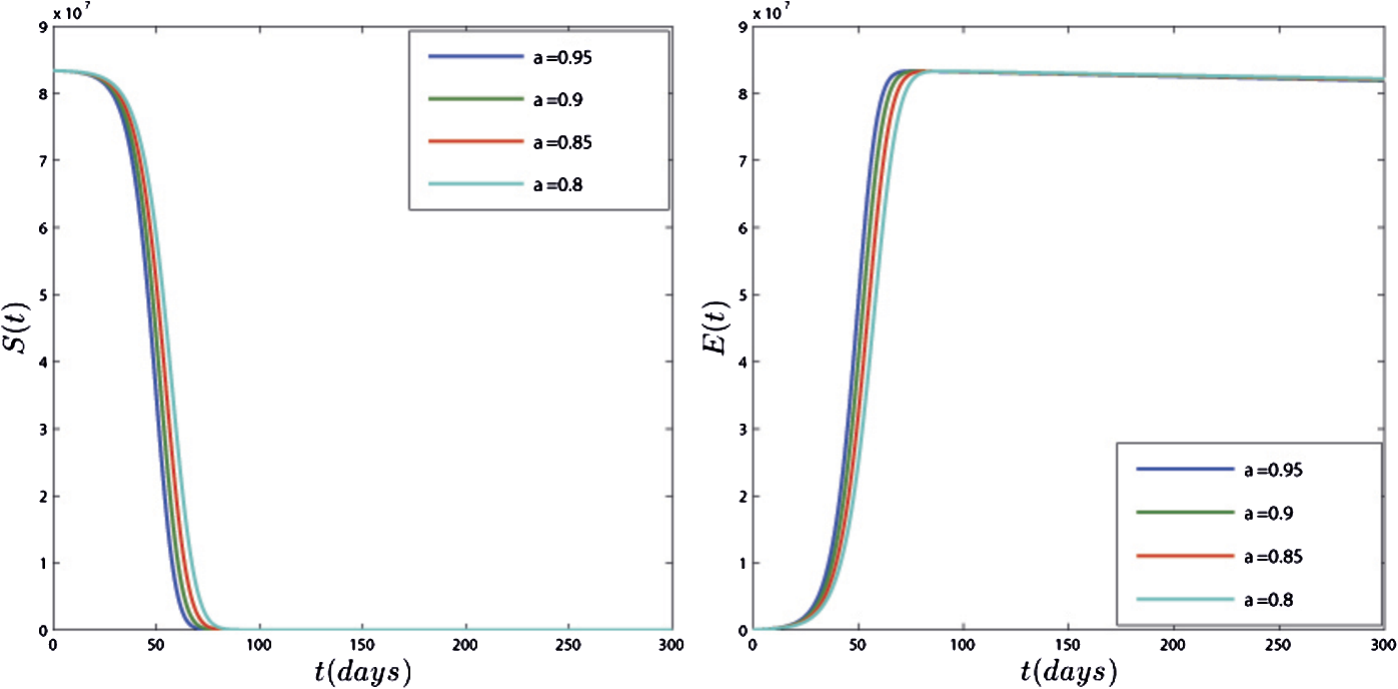
Plots of $S(t)$ and $E(t)$ for different values of $\nu =0.95, 0.9, 0.85, 0.8$

**Figure 8:**
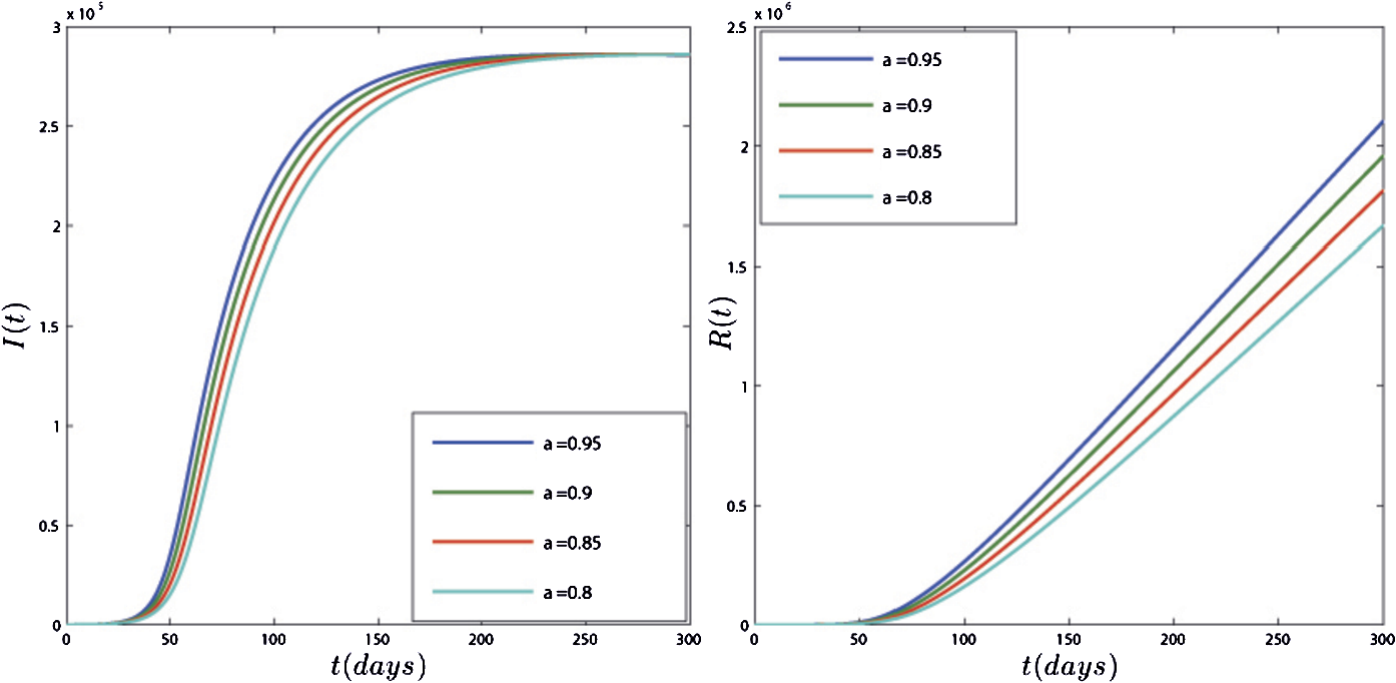
Plots of $I(t)$ and $A(t)$ for different values of $\nu =0.95, 0.9, 0.85, 0.8$

In the second case, we provide a numerical simulation using real data for the transmission model of COVID-19 in Iran. According to the WHO report, the total population of Iran on 18 February 2020 was $N=83392425$, the birth rate for Iran in 2019 was 18.547 births per 1000 people, and the death rate was 4.866 per 1000 people. Thus, for every day, we have $\omega =\frac{n \times N}{365}=4237.477$ and $\mu =\frac{0.004866}{365}=0.0000133315$. Similarly, we assumed the mortality rate due to the disease in Iran is $\delta =3.4\times 10^{-2}$ and $\theta =0.99$. Since $N(0)=S(0)+E(0)+I(0)+R(0)$ and on 18 February we have $I(0)=61$, $R(0)=12$, then we assume $E(0)=3000$ and $S(0)=83\mbox{,}389\mbox{,}352$. For the fitting, we use the information provided by the World Health Organization for COVID-19. The fitted curve and the reported cases of COVID-19 in Iran from 18 February to 12 April 2020 are plotted in Fig. 6, so that every part is three days. Using this method, we obtain the parameters as follows: $\beta _{1}=1.1\times 10^{-4}$, $\beta _{2}=3.3\times 10^{-6}$, $\lambda =1.02 \times 10^{-3}$, $\tau = 0.03$. In Figs. 7 and 8, we plotted the results of the system of COVID-19 transmission (1). As you can see in Figs. 7 and 8, the variables have different results in different amounts of *ν* but exhibit the same behavior. Figure 7 shows that two months after the virus is released almost the entire population is at risk for the disease. Figure 8 shows that the number of people with COVID-19 increases until 200 days and then stabilizes. Also, the forecast is that the number of infected people could rise to 280,000. Also, Fig. 8 shows that the number of people who have recovered or died also increases over time.

## Conclusion

In this work, the SEIR epidemic model for the transmission of COVID-19 using the Caputo fractional derivative has been presented. The feasibility region of the system and equilibrium points have been calculated, and the stability of the equilibrium points has been investigated. The existence of a unique solution for the model by using fixed point theory has been proved. Using the fractional Euler method, an approximate answer to the model has been calculated. To predict the transmission of COVID-19 in the world and in Iran, the numerical simulations based on real data have been provided. Also in the numerical section, we have examined the advantage of using the fractional-order derivative instead of the integer-order one, and in Table 1 and Fig. 3, we have compared the results of the model with the fractional- and integer-order derivative and the real data. The results show that the fractional-order model has a better result in this modeling.
